# ABO blood group frequency in Ischemic heart disease patients in Pakistani population

**DOI:** 10.12669/pjms.303.4502

**Published:** 2014

**Authors:** Saima Sharif, Naureen Anwar, Tasnim Farasat, Shagufta Naz

**Affiliations:** 1 Dr. Saima Sharif, Assistant Professor, Department of Zoology, Lahore College for Women University, Lahore, Pakistan.; 2 Naureen Anwar, MS student, Department of Zoology, Lahore College for Women University, Lahore, Pakistan.; 3 Tasnim Farasat, Professor, Department of Zoology, Lahore College for Women University, Lahore, Pakistan.; 4 Dr. Shagufta Naz, Assistant Professor, Department of Zoology, Lahore College for Women University, Lahore, Pakistan.

**Keywords:** ABO blood group, Ischemic heart disease

## Abstract

***Objectives:*** To determine if there is any significant association between ABO blood groups and ischemic heart disease (IHD).

***Methods:*** The study was performed at Punjab Institute of Cardiology (PIC), Lahore. Study duration was from January 2012 to September 2012. This study included 200 IHD patients and 230 control individuals. Self design questionnaire was used to collect information regarding risk factors. Standard agglutination test was performed to determine the blood groups. Data was analyzed on SPSS 16.

***Results: ***The prevalence of blood groups in IHD group was 34% in blood group A, 29% in blood group B, 14% in blood group AB and 23% in blood group O. In control group the distribution of B, A, AB and O blood groups were 34.4%, 20.9%, 12.6%, 32.2% respectively. Rh+ve factor was prevalent in 90.5% among IHD group and 92.6% in control subjects. The prevalence of IHD was more in males (63.5%) as compared to females (36.5%). Mean age was 56.4±0.86 (yrs) and BMI was 26.4±0.33 (kg/m^2^). The prevalence of hypertension was 58.5%, diabetes was 53%, family history of cardiac disease was 45%, 35.5% of patients were doing exercise regularly, 58.5% used ghee, and 58% were smokers.

C***onclusion:*** Subjects with blood group A had significantly (p< 0.05) higher risk of developing IHD as compare to other blood groups.

## INTRODUCTION

IHD is one of the most critical problems of the civilized world. Of the 16.7 million deaths from CVDs every year, 7.2 million are due to IHD.^1^

During past years, many reports have appeared showing association of blood groups to coronary heart disease and IHD. Among different categories of IHD, the frequencies of stable angina, acute myocardial infarction, and stable angina were higher in AB blood group.^2^ In UK population IHD was found to be prevalent in AB blood group.^3^ Wazirali showed blood group A was 3.14 fold more prevalent than blood group B, 6.35 fold than blood group O, and 3.32 fold than blood group AB.^4^ Similarly blood group A was dominant in patients in Rawalpindi.^5^

The present study was designed to investigate the distribution of blood groups in IHD in our local population.

## METHODS

The study was carried out at Punjab Institute of Cardiology, Lahore. Before conducting this research, approval was taken from ethical committee of hospital and a written consent was taken from each participant. A total of 430 samples were collected and divided into two groups i.e. control (n= 230) and IHD subjects (n=200). Controls were collected from current blood donors and individuals having normal electrocardiogram (ECG).

A questionnaire was filled by each subject to collect information regarding age, gender, hypertension, diabetes, family history of ischemic heart disease, regular exercise, use of ghee or oil for cooking food, and smoking status. Weight was also measured with weighing machine in kg. Height was measured by measuring tape. BMI was calculated by using the formula:^6^

BMI = weight (kg) / height (m^2^)

Blood samples were collected and blood group was determined by agglutination method using antisera A, B, and D.Data was analyzed using SPSS 16. Demographic data was presented as Mean ± SEM. T- test was used for comparison of continuous variables. Chi-Square (X^2^) was used for non-discrete variables. P-value less than 0.05 were taken as significant.

## RESULTS

The control group, which comprised of 230 subjects, includes 167 (72.6%) males and 63 (27.4%) females. The IHD group consists of 127 (63.5%) males and 73 (36.5%) females.

The major risk factors found in our study were smoking, diabetes, hypertension, sedentary life style, and the use of ghee for cooking food. They are suspected to increase the chances for the development of IHD.

**Table-I:** Frequency of risk factors in IHD group

*Sr. No*	*Risk factors*	*Yes*	*No*
1	Smoking	108 (54%)	92 (46%)
2	Family history	90 (45%)	110 (55%)
3	Diabetes	107 (53.5%)	93 (46.5%)
4	Hypertension	117 (58.5%)	83 (41.5%)
5	Exercise	71 (35.5%)	129 (64.5%)
6	Use of ghee	117 (58.5%)	83 (41.5%)

**Table-II:** Demographic characteristics of control and IHD group

*Sr. No*	*Factors*	*Control*	*IHD*	*P value (T – Test)*
1	Age (years)	34.3 ± 0.71	56.4 ± 0.86	**0.001** **
2	Weight (kg)	63.5 ± 0.59	69.2 ± 0.87	**0.001** **
3	Height (m)	1.64 ± 0.06	1.61 ± 0.03	**0.05** *
4	BMI (kg/m^2^)	23.8 ± 0.29	26.4 ± 0.33	**0.001** **

* Significant P ≤ 0.05,

** Highly significant P ≤ 0.01

**Table-III:** Prevalence of blood groups in control and IHD groups

*Sr. no*	*Blood groups*	*Control group (%)*	*IHD group (%)*	*Percentage*			
				*Rh+*		*Rh-*	
				*Control*	*IHD*	*Control*	*IHD*
1.	A	20.9	34	19.1	32	1.8	2
2.	B	34.4	29	32.2	24	2.1	5
3.	AB	12.6	14	12.6	13.5	-	0.5
4.	O	32.2	23	28.7	21	3.5	2

According to the study the distribution of A, B, AB, O blood groups in the control group were as follow, 48 (20.9%) had blood group A, 79 (34.3%) had blood group B, 29 (12.6%) had blood group AB, and 74 (32.3%) had blood group O. In IHD group, 68 (34%) had blood group A, 58 (29%) had blood group B, 28 (14%) had blood group AB, and 46 (23%) had blood group O.

The order of percentage of ABO blood groups among control group was found to be in order B>O>A>AB and the order of percentage for Rhesus factor was Rh +ve> Rh –ve. The order of percentage of ABO blood groups among IHD group was found to be in order A>B>O>AB and the order of the percentage for Rhesus factor in patients was Rh +ve> Rh –ve. It was found that the prevalence of blood group A was high among IHD group i.e. 34%. The high percentage of blood group A (p-value < 0.05) shows that there is a strong relation of blood group A with IHD.

## DISCUSSION

In different regions of the world there is specific ABO blood group distribution. Even in the same country as in Pakistan minor variations has been observed.^7^^,^^8^ In Sindh and Baluchistan blood group O is more common in normal population.^9^

A significant association was found in Italian population between blood groups and family history of IHD and were associated with increased mortality in patients.^10^ Anvari showed CABG patients in Iranian population have high prevalence of blood group A.^11^ In a British regional heart study, 7735 men with IHD were examined showing that blood group A is associated with IHD in middle aged British men.^12^

The results of our study showed a significant association (p-value < 0.05) between IHD and blood group A. In control group, blood group B had higher prevalence (34.4%).

Framingham Heart study in 1948 showed that many different parameters were associated with the development of IHD. With the development of modern science certain parameters became recognized as risk factors of IHD.^13^ Age, sex, family history of IHD and height are non-modifiable risk factors Smoking, hypertension, diabetes mellitus, obesity are major modifiable risk factors.^14^^, ^^15^^, ^^16^ Obese people are at greater risk to suffer from CVDs.^17^

This study clearly indicates a high prevalence of risk factors in IHD group as compared to the control group. The mean BMI greater in IHD as compared to control group indicates that majority of IHD subjects were obese and it can be predicted that obesity may play an important role in developing IHD in our local population.

Smoking is the single most important modifiable risk factor for CVDs and the leading preventable cause of death.^18^ Compared with non- smokers, those who consume 20 or more cigarette daily have twofold to threefold increase in total coronary heart disease.^19^ It has been recommended that the constituents of ghee consist of a probable cause of high CHD risk among South Asian including Pakistanis.^20^

The ratio of smoking and use of ghee for cooking food was found as 58% and 58.5% respectively. Family history was found to be insignificant in IHD group with a percentage of 45%. 

## CONCLUSION

The results of the present study revealed a significant association of blood group A with IHD. Risk factors like smoking, age, obesity, use of ghee, and lack of doing exercise was found to be more prevalent in IHD group. Thus these might be the major contributing factors for developing the risk of IHD in our local population.

## Authors Contributions:

**SS:** Conceived and designed the protocol of the project, contributed in writing of the manuscript.

**NA:** Did data collection, experimental work and writing of manuscript.

**TF:** Contributed in writing of manuscript, did editing and review of manuscript.

**SN:** Contributed in statistical analysis.
